# Glycyrrhetic Acid Synergistically Enhances β_2_-Adrenergic Receptor-Gs Signaling by Changing the Location of Gαs in Lipid Rafts

**DOI:** 10.1371/journal.pone.0044921

**Published:** 2012-09-27

**Authors:** Qian Shi, Yuanyuan Hou, Jie Hou, Penwei Pan, Ze Liu, Min Jiang, Jie Gao, Gang Bai

**Affiliations:** 1 College of Pharmacy, State Key Laboratory of Medicinal Chemical Biology and Tianjin Key Laboratory of Molecular Drug Research, Nankai University, Tianjin, China; 2 College of Life Sciences, Nankai University, Tianjin, China; 3 College of Medicine, Nankai University, Tianjin, China; University of Oldenburg, Germany

## Abstract

Glycyrrhetic acid (GA) exerts synergistic anti-asthmatic effects via a β_2_-adrenergic receptor (β_2_AR)-mediated pathway. Cholesterol is an important component of the structure and function of lipid rafts, which play critical roles in the β_2_AR-Gs-adenylate cyclase (AC)-mediated signaling pathway. Owing to the structural similarities between GA and cholesterol, we investigated the possibility that GA enhances β_2_AR signaling by altering cholesterol distribution. Azide-terminal GA (ATGA) was synthesized and applied to human embryonic kidney 293 (HEK293) cells expressing fusion β_2_AR, and the electron spin resonance (ESR) technique was utilized. GA was determined to be localized predominantly on membrane and decreased their cholesterol contents. Thus, the fluidity of the hydrophobic region increased but not the polar surface of the cell membrane. The conformations of membrane proteins were also changed. GA further changed the localization of Gαs from lipid rafts to non-raft regions, resulting the binding of β_2_AR and Gαs, as well as in reduced β_2_AR internalization. Co-localization of β_2_AR, Gαs, and AC increased isoproterenol-induced cAMP production and cholesterol reloading attenuated this effect. A speculation wherein GA enhances beta-adrenergic activity by increasing the functional linkage between the subcomponents of the membrane β_2_AR-protein kinase A (PKA) signaling pathway was proposed. The enhanced efficacy of β_2_AR agonists by this novel mechanism could prevent tachyphylaxis.

## Introduction

Lipid rafts are small (10 nm to 200 nm), heterogeneous, highly dynamic, cholesterol and sphingolipid-enriched microdomains of plasma membranes [1]. Numerous membrane proteins are known to partition into lipid rafts. Such proteins include glycosylphosphatidyl inositol (GPI)-anchored proteins [2], [3], doubly acylated proteins, such as Src-family kinases and the α-subunits of heterotrimeric G proteins [4], cholesterol-linked proteins, and palmitoylated proteins [5]. Lipid rafts have been implicated in the regulation of various cell signaling pathways, and recent studies have indicated that many multi-component signaling pathways are coordinated by co-localization in lipid rafts, including the immunoglobulin E [6], [7], T-cell antigen receptor [8], [9], Ras [10], and G protein-coupled receptor (GPCR) [11] signaling pathways. While the functional significance of lipid rafts has not been completely understood, the rafts appear to enrich receptors and effectors, thus facilitating the interaction of signaling components, as in serotonin-1A receptor and neurokinin-1 receptor signaling regulation [12], [13]. However, despite the positive role of lipid rafts, raft disruption can also enhance signal transduction through epidermal growth factor receptors [14], revealing a potential negative regulatory role in signal transduction.

Cholesterol serves as a spacer between the saturated chains of sphingolipids and is essential for maintaining the liquid-ordered phase of rafts and segregating embedded proteins from the rest of the membrane [15], [16]. Small changes in the plasma membrane cholesterol content near the physiological set point may alter a variety of large biological responses [17], [18]. Accumulating evidence also suggests that membrane cholesterol could impinge on signal transduction by directly affecting receptor affinity [11] and protein activity via sterol-sensing domains [19]. In the canonical signaling pathway mediated by seven transmembrane β_2_-adrenergic receptors (β_2_AR), cholesterol depletion was found to increase β_2_AR-stimulated cAMP production [20], [21]. Cholesterol supplementation has an inverse effect on such production [22]. Some natural cholesterol derivatives, including steroid hormones, phytosterols, or saponins, can also alter signal transduction by changing the integrity of lipid rafts [23], [24]. Considering the properties of plasma membrane, cholesterol or cholesterol-like substances may play important roles in signal transduction.

Liquorice, one of the best-known herbaceous plants, has been widely used in traditional Chinese medicine for thousands of years. Glycyrrhizin, a triterpene saponin and the major pharmacologically active compound in liquorice, possesses various pharmacological effects, such as anti-inflammatory [25], anti-viral [26], [27], and anti-carcinogenic activities [28]. Glycyrrhizin also exhibits anti-allergic effects and has been demonstrated to be useful for treating airway and lung disorders [29], [30]. In a previous study, we demonstrated that co-treatment with glycyrrhizin and β_2_AR agonists yields synergistic anti-asthmatic effects, and that glycyrrhizin enhances β_2_AR signaling by increasing β_2_AR agonist-stimulated cAMP accumulation [31]. Pharmacological studies have revealed that after oral administration, glycyrrhizin is hydrolyzed to glycyrrhetic acid (GA), the aglycone of glycyrrhizin, by human intestinal bacteria prior to absorption through a specialized β-glucuronidase [32]. However, GA has higher bioavailability than glycyrrhizin [33], and whether or not GA has functions similar to glycyrrhizin remains to be determined.

In this study, we investigated the underlying mechanism of GA regulatory effect on β_2_AR-Gαs-adenylate cyclase (AC) signal transduction. GA was demonstrated to change the localization of Gαs from lipid rafts to the non-raft region. The results show GA reduced level of β_2_AR internalization, thereby increasing downstream cAMP production. The effect of GA on β_2_AR-Gαs-AC signaling is linked to its ability to decrease the cholesterol content of lipid. This effect is evidenced by the finding that the targeting of a GA probe to membrane and enhanced β_2_AR-Gαs-AC signaling are strongly attenuated by cholesterol reloading.

## Materials and Methods

### Cell Lines and Chemosynthesis

Hemagglutinin (HA)-β_2_AR HEK293 cells were derived from HEK293 cells. β_2_ARs were expressed with an N-terminal HA epitope tag. The enhanced green fluorescent protein-β_2_AR (β_2_AR-EGFP) plasmid was constructed in our laboratory [31]. The synthesis experimental procedures of the compounds azide-terminal GA (ATGA), 4-ethynyl-N-ethyl-1, 8-naphthalimide and linker-biotin are described in the Supporting Information files.

### Analysis of Fluorescent Labeling in Cells by Confocal Microscopy

HA-β_2_AR HEK293 cells were cultured and supplied with 0.1 mM GA (J&K Chemical, Beijing, China) or 0.1 mM ATGA for 1 h. After fixing with 4% paraformaldehyde, the cells were subjected to a probe labeling reaction (0.1 mM linker-biotin, 0.1 mM Tris-triazoleamine, 0.1 mM CuSO_4_, and 1 mM sodium ascorbate in phosphate-buffered saline, PBS) at room temperature for 30 min. Subsequently, the cells were labeled with mHA.11 antibody (Covance, CA, USA) and visualized using fluorescein isothiocyanate-conjugated secondary antibody and Cy3-conjugated streptavidin. Fluorescent images were captured by a LEICA TCS SP5 laser scanning confocal microscopy system (Leica Microsystems, Heidelberg, Germany).

For live cell microscopic imaging, HA-β_2_AR HEK293 cells were treated with GA or ATGA. The reaction was carried out using 4-ethynyl-*N*-ethyl-1, 8-naphthalimide with sodium ascorbate, CuSO_4_, and Tris-triazoleamine catalyst as described above (wavelength: excitation = 365 nm, emission = 465 nm).

### Detergent-free Purification of Lipid Raft Membrane Fractions

The detergent-free purification of cholesterol-enriched microdomains was carried out according to the protocol described by Song et al. [34]. After two washes in cold PBS, HA-β_2_AR-HEK293 cells treated with or without 0.1 mM GA for 2 h were scraped into 500 mM sodium carbonate (pH 11.0) and homogenized by sonication. The homogenate was adjusted to 40% sucrose, placed at the bottom of an ultracentrifuge tube (Beckman Instruments), and overlaid with a 5% to 35% discontinuous sucrose gradient.

### Immunoblotting

Equal volumes of each gradient fraction were loaded to 10% SDS-polyacrylamide gel electrophoresis. For immunodetection, the following antibodies were used: rabbit polyclonal antibody against anti-β_2_AR (clone H-20), anti-flotillin-1 (clone H-104), and anti-Gαs (clone K-20) (all from Santa Cruz Biotechnology, CA, USA). The rabbit polyclonal antibody against caveolin-1 was from Abcam Inc. (Cambridge, MA, USA).

### Cholesterol and ATGA Assays

The cholesterol content of the sucrose gradient fractions was measured using a Cholesterol/Cholesteryl Ester Quantitation kit (Biovision, CA, USA). Each sucrose gradient fraction was extracted with chloroform/isopropanol/nonyl phenoxypolyethoxylethanol (NP-40) (7∶11:0.1). The lipids were extracted, and all traces of organic solvents were evaporated prior to resuspending the lipids in the cholesterol assay buffer. The assays were performed according to the manufacturer’s instructions. For the analysis of ATGA content, the samples were prepared as for cholesterol analysis, except that the assay buffer contained 0.1 mM 4-ethynyl-*N*-ethyl-1,8-naphthalimide, 0.1 mM Tris-triazoleamine catalyst, 0.1 mM CuSO_4_, and 1 mM sodium ascorbate. Then, the reaction mixture was transferred to 96-well plates and scanned by a Thermo Scientific Varioskan Flash spectral scanning multimode reader (Varioskan, Thermo Electron Co, MA, USA) with SkanIt software.

### Filipin Fluorescence Staining of Cell Membrane Cholesterol

HA-β_2_AR HEK293 cells were cultured with or without 0.1 mM GA for 2 h at 37°C. After fixing with 4% paraformaldehyde, the cells were stained with 1 ml of a filipin working solution (0.05 mg/ml in PBS with 10% fetal bovine serum) for 2 h. Images were obtained under a LEICA TCS SP5 laser scanning confocal microscopy system (Leica Microsystems, Heidelberg, Germany) using a UV filter set.

### Spin Labeling of Cells with 5-doxyl Stearic Acid (5-DSA), 16-doxyl Stearic Acid (16-DSA), and 3-maleimide-proxyl (3-MP)

The spin labels 5-DSA and 16-DSA consist of a radical group (doxyl) and a hydrocarbonated chain (stearic acid) that acts as a radical support. Since spin labels are oriented and linked like the lipids in the lipid bilayers of cell membranes. These nitroxyl radicals in the 5th or 16th positions of the alkyl chain can be used to determine local fluidity in the two main regions of the cell membrane, the region near the polar head group (5-DSA), and the hydrophobic region (16-DSA) within the lipid bilayers.

After treatment with 0.1 mM GA, the HA-β_2_AR HEK293 cell suspension was mixed with spin label 5-doxyl or 16-docyl (30 µg/ml) and incubated at 37°C for 30 min. The free spin labels were washed out by PBS from the cell suspension until no signal was detected in the supernatant. The electron spin resonance (ESR) measurement conditions were as follows: microwave power, 20 mW; modulation frequency, 100 kHz; modulation amplitude, 2 G; sweep width, 100 G; and temperature, 24°C. The parameter *S* and rotational correlation time *τ*_c_ are defined as follows:
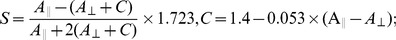


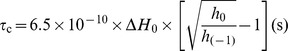
where *h*_0_ and *h*_(−1)_ are the peak heights of the center and high field lines, respectively, Δ*H*_0_ is the width of the central line; and *A*_∥_ and *A*_⊥_ are the parallel and perpendicular hyperfine splitting parameters of the spectrum, respectively.

For 3-MP labeling, cells treated with GA were labeled by 3-MP (20 µg/ml) and incubated at 37°C for 3 h. Then, the suspension was washed in PBS until no ESR signal was detected in the supernatant. The measurement conditions were as follows: microwave power, 20 mW; modulation frequency, 100 kHz; modulation amplitude, 1 G; sweep width, 300 G; and temperature, 24°C.

### Co-immunoprecipitation of β_2_AR with β-arrestins and Clathrin

Confluent cells were scraped into ice-cold lysis buffer (pH 7.4 50 mM Tris-HCl, 150 mM NaCl, 1 mM EDTA, 0.1% SDS, 1% sodium deoxycholate, 1% Triton X-100, and 1 mM phenylmethylsulfonylfluoride) and incubated for 3 h at 4°C. The total cellular protein mixture was centrifuged at 21 000×*g* for 15 min. The supernatant (detergent-soluble extract) was then mixed with 20 µL of an anti-HA affinity matrix (Roche Diagnostics, Indianapolis, IN, USA) at 4°C overnight. After washing with ice-cold lysis buffer, the immune complexes were boiled in SDS sample loading buffer and subjected to electrophoresis on a 4–20% Tris-glycine gel. Immunoblotting was executed with anti-β-arrestins or clathrin (Santa Cruz, San Diego, CA, USA) heavy chain polyclonal antibody.

### Live Cell Imaging Microscopy for Receptor Internalization Observation

To monitor the internalization of β_2_ARs upon receptor activation, HEK293 cells were transiently transfected with the β_2_AR-EGFP plasmid. The cells with or without 0.1 mM GA pretreatment were stimulated with 0.2 mM isoproterenol (Sigma, St. Louis, MO, USA) for up to 30 min. β_2_AR-EGFP fluorescent signals were recorded under a LEICA TCS SP5 confocal laser scanning microscope (Leach Instruments, Heidelberg, Germany). The excitation and emission wavelengths employed were 488 and 520 nm, respectively. Sequential confocal sections (z plane) were acquired at intervals of 0.5 µm from the middle to the bottom of the cells.

### Fixed-cell ELISA Assays for Cell Surface β_2_AR Detection

HA-β_2_AR-HEK 293 cells growing on 96-well microplates were exposed to 0.1 mM salbutamol for up to 2 h at 37°C, fixed with 4% paraformaldehyde, and blocked with 4% skimmed milk. Antibody mHA.11 and HRP-labeled second antibody were used to detect HA-β_2_AR. Finally, the content of HA-β_2_AR was determined by a 3, 3′, 5, 5′-tetramethylbenzidine solution.

### Luciferase Reporter Assay for cAMP Accumulation

Cells were co-transfected with pCRE-Luc reporter plasmid and pRL-TK plasmid (Promega, Madison, USA). After 24 h, cells were treated with different concentrations of the indicated drugs for 5 h. In the glycyrrhizin pretreatment group, cells were pretreated with 0.1 mM GA for 3 h, and the reaction was stopped by washing with PBS buffer. The cells were lysed and the luciferase activity was measured using a Dual-Luciferase reporter assay system (Promega). Luminescence was detected using a Modulus Luminometer from Turner Biosystems (Turner design, Sunnyvale, CA, USA). The ratio of firefly luciferase activity to renilla luciferase activity was used to normalize for differences in the transfection efficiency.

### Statistical Evaluation

Data were expressed as mean ± S.E.M. of triplicate determinations from at least three independent experiments. Statistical analysis was performed using Student’s *t*-test or two-way analysis of variance (ANOVA). *p*<0.05 was considered statistically significant.

## Results

### Cellular Localization of ATGA

**Figure 1:**
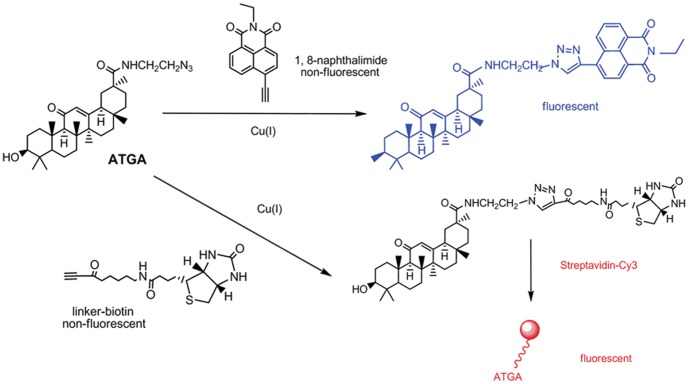
Synthesis of ATGA and two strategies for GA labeling. Probe structures based on 4-ethynyl-*N*-ethyl-1,8-naphthalimide or biotin linked alkyne will allow a fluorogenic ligation with ATGA. The fluorescence adduct is generated when probes are reacted with the azido group of GA via Cu (I)-catalyzed [3+2] cycloaddition.

To identify the cellular targets of GA, we prepared an ATGA probe. The synthesis of ATGA is outlined in Materials S1. Owing to the azido modification, ATGA could undergo a Cu (I)-catalyzed [3+2] cycloaddition reaction with an alkyne group (Fig. 1) [35].

**Figure 2:**
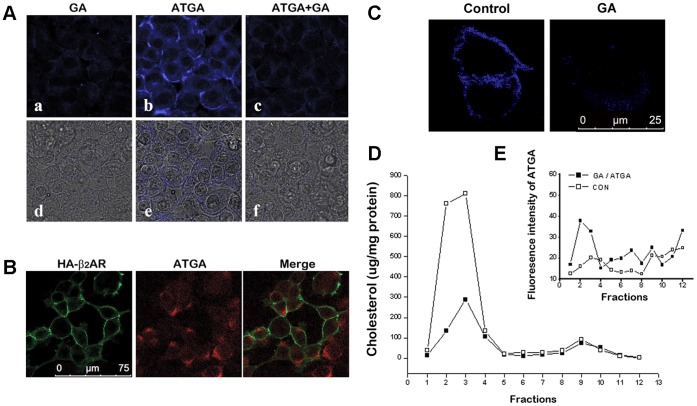
Displacement effect of GA in HA-β_2_AR-HEK293 cells. (A) HA-β_2_AR-HEK293 cells were treated with (a) 10 µM GA, (b) ATGA, or (c) a mixture of 100 µM GA and 10 µM ATGA. Live cells were imaged. (d to f) show the merged images of UV and light of (a to c), respectively. (B) Analysis of ATGA and HA-β_2_ARs co-localization by confocal fluorescence microscopy. (C) β_2_AR-HEK293 cells treated with or without GA were stained with filipin (blue) for detection of cholesterol. Triton X-100 extracts of HA-β_2_AR-HEK293 cells were run on sucrose gradients, and the separated gradient fractions were assayed for the distribution of total cholesterol (D) and ATGA (E), as described in the Methods section.

Fig. 2A shows the location of ATGA. The blue fluorescence is clearly concentrated on the cell membrane (b and e). In contrast, only a low level of background fluorescence was observed in the GA-treated control group (a and d), supporting the specificity of the click-chemistry reactions between the azide (ATGA) and alkyne groups (4-ethynyl-*N*-ethyl-1,8-naphthalimide), which is synthesized through the process described in Materials S2. The pretreatment of cells with GA significantly inhibited the uptake of ATGA, indicating that ATGA and GA compete for the same cellular targets (c and f). These observations suggest that ATGA mainly localizes in the cell membranes and provides a suitable mimic for GA. Furthermore, a fluorescence microscopy of ATGA and β_2_AR was performed. ATGA was reacted with biotin linked alkyne (Materials S3) and visualized by Cy3 labeled strepavidin (red) and the β_2_ARs were visualized with a proper antibody (green). The week co-location of ATGA and β_2_AR shows in Fig. 2B indicated that ATGA and β_2_AR may exist in different microdomains.

### GA Decreased the Cholesterol Content of Lipid Rafts

The staining of cholesterol (Fig. 2C) shows the effect of GA on cholesterol localization. As cholesterol is the primary content of lipid rafts, it was mainly detected on cell membrane. Under GA treatment, cholesterol was dispersed as a much weaker signal compared with the control experiment. To confirm the decrease effect of GA on cholesterol on cell membrane, we evaluated the cholesterol content in different cell fractions. Triton X-100 sucrose-gradient fractions were analyzed for total protein, cholesterol, and ATGA contents. As shown in Fig. 2D, the bulk of the cholesterol was found in the low-density fractions at the upper end of the gradient (fractions 2 to 4) which should correspond to the lipid rafts distribution, and the content significantly decreased when treated with GA. The distribution of ATGA was also determined by reaction with 4-ethynyl-*N*-ethyl-1, 8-naphthalimide. The fluorescence intensity indicated that ATGA distributes predominantly in light-density fractions (fractions 2 to 4), which also correspond to cholesterol (Fig. 2E). This result indicates that the ATGA might decrease the cholesterol on membrane by a displacement effect.

### GA Changed the Fluidity of Cell Membranes

**Figure 3:**
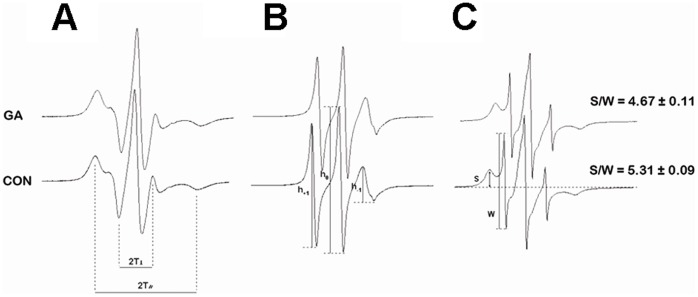
ESR spectra analysis for cell membranes fluidity of HA-β_2_AR-HEK293 cell. Spin labeling of cell with 5-DSA (A), 16-DSA (B), or 3-MP (C).

Cholesterol is a major determinant of membrane fluidity. Previous findings suggest that GA displacement alters the membrane fluidity of lipid rafts similar to other cholesterol derivatives [36]. ESR was utilized to investigate the effect of GA on membrane fluidity and membrane protein conformations. The ESR spectra of spin-labeled membranes with 5-DSA and 16-DSA are shown in Fig. 3A and Fig. 3B. The order parameter *S* and rotational correlation time *τ*_c_ calculated from the spectra of 5-DSA and 16-DSA were 0.6551±0.003 and 16.03±0.11×10^−10^ (s), respectively. Compared with the control group, the order parameter remained almost unchanged after GA treatment (0.6653±0.005), whereas *τ*_c_ significantly decreased after GA treatment (15.13±0.15, *p*<0.05). This result indicates that GA significantly increases lipid fluidity in the hydrophobic region of plasma membranes.

To identify change in the properties of membrane proteins, 3-MP spin labels that can specifically bond with the sulfhydryl groups of the proteins were employed. GA treatment decreased the *S*/*W* ratio from 5.31±0.09 in the control HA-β_2_AR-HEK293 cells to 4.67±0.11 after treatment with 0.1 mM GA (*p*<0.05). This change in *S*/*W* indicates that the structures of sulfhydryl binding sites on membrane proteins become tighter after GA treatment owing to conformational changes caused by GA. The results reveal that GA treatment influences the fluidity in the hydrophobic regions of cell membranes, and thereby alters the conformations of many membrane proteins.

### GA Changed the Localization of Signaling Molecules Gα_s_ and Increased β_2_AR/G Protein Coupling in Plasma Membrane

**Figure 4:**
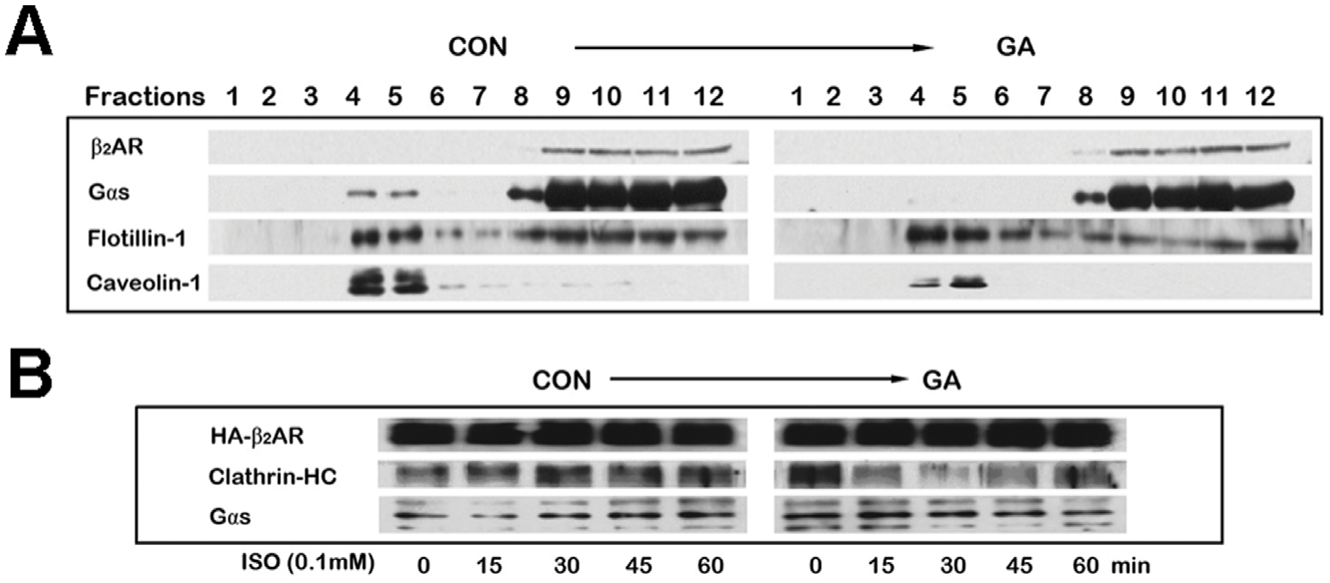
Effect of GA on the distribution of signaling molecules and β_2_AR/G protein coupling in plasma membrane. (A) Lipid raft fractions were purified following a sodium carbonate detergent-free method on a sucrose gradient. The presence of β_2_AR, Gα_s_, flotillin-1, and caveolin-1 was detected in each fraction by Western blot analysis using the appropriate antibodies. (B) Co-immunoprecipitation of Gα_s_ and clathrin heavy chains with HA-β_2_AR. GA decreased β_2_AR binding to clathrin but increased β_2_AR binding to Gα_s_. Cells were pretreated with 0.1 mM GA, followed by 0.1 mM isoproterenol stimulation for the indicated time. An anti-HA affinity matrix was used to precipitate HA-β_2_AR. Co-immunoprecipitated endogenous Gα_s_ and clathrin heavy chain were detected by Western blot analysis.

We investigated whether or not GA could change the localization of signaling molecules in lipid rafts. Membranes from GA-treated and control HA-β_2_AR-HEK293 cells were fractionated by the sodium carbonate procedure. The signaling molecules present in the fractions were then determined by immunoblotting (Fig. 4A) [34]. The lipid raft marker molecule flotillin and the scaffold protein of caveolae, caveolin-1, were mainly detected in fractions 4 and 5, which are considered to containing lipid raft fractions. The G-protein subunit Gα_s_ was abundant in the lipid raft fractions from control cells. Treatment of cells with GA resulted a significant loss of Gα_s_ in lipid raft fractions, this part of Gα_s_ might translocated to high-density fractions (fractions 8–12), whereas the distributions of flotillin and caveolin-1 were not affected.

Co-immunoprecipitation experiments were performed to identify the G proteins associated with β_2_ARs. Western blot analysis of β_2_AR immunoprecipitations revealed that Gα_s_ was preassociated with the receptor under the basal condition in untreated cells (Fig. 4B, left panel, 0 min). Upon the stimulation of isoproterenol, Gα_s_ rapidly separated from β_2_AR (Fig. 4B, left panel, 15 min). When the cells were pretreated with GA, we found a significant increase in the association of G proteins with unstimulated β_2_AR (Fig. 4B, 15 min). No significant reduction in the amount of these β_2_AR/Gα_s_ complexes was observed after 15 min of isoproterenol stimulation.

### GA Pretreatment Significantly Inhibited the Internalization of β_2_AR

**Figure 5:**
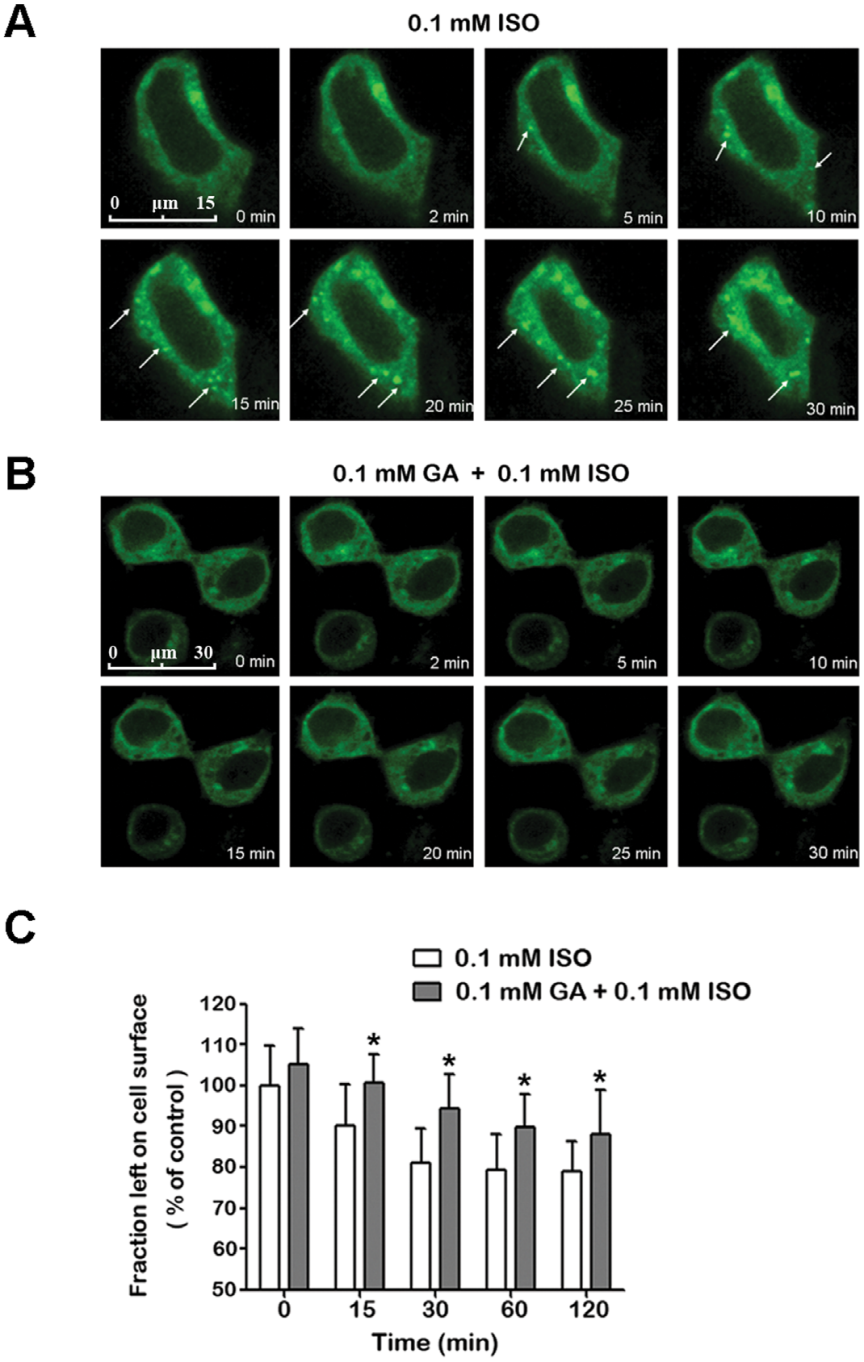
GA pretreatment significantly inhibited internalization of β_2_AR. The subcellular distribution of EGFP-tagged β_2_ARs upon 0.1 mM ISO stimulation (A) without or (B) with 0.1 mM GA pretreatment. The arrow indicates the internalization of β_2_AR. (C) Quantification of surface β_2_ARs using fixed HA-β_2_AR-HEK 293 cell ELISA with Anti-HA tag mAb. The time course comparison reveals a difference between groups with or without GA pretreatment as assessed by two-way ANOVA. *means *p*<0.05 (*n* = 3).

**Figure 6:**
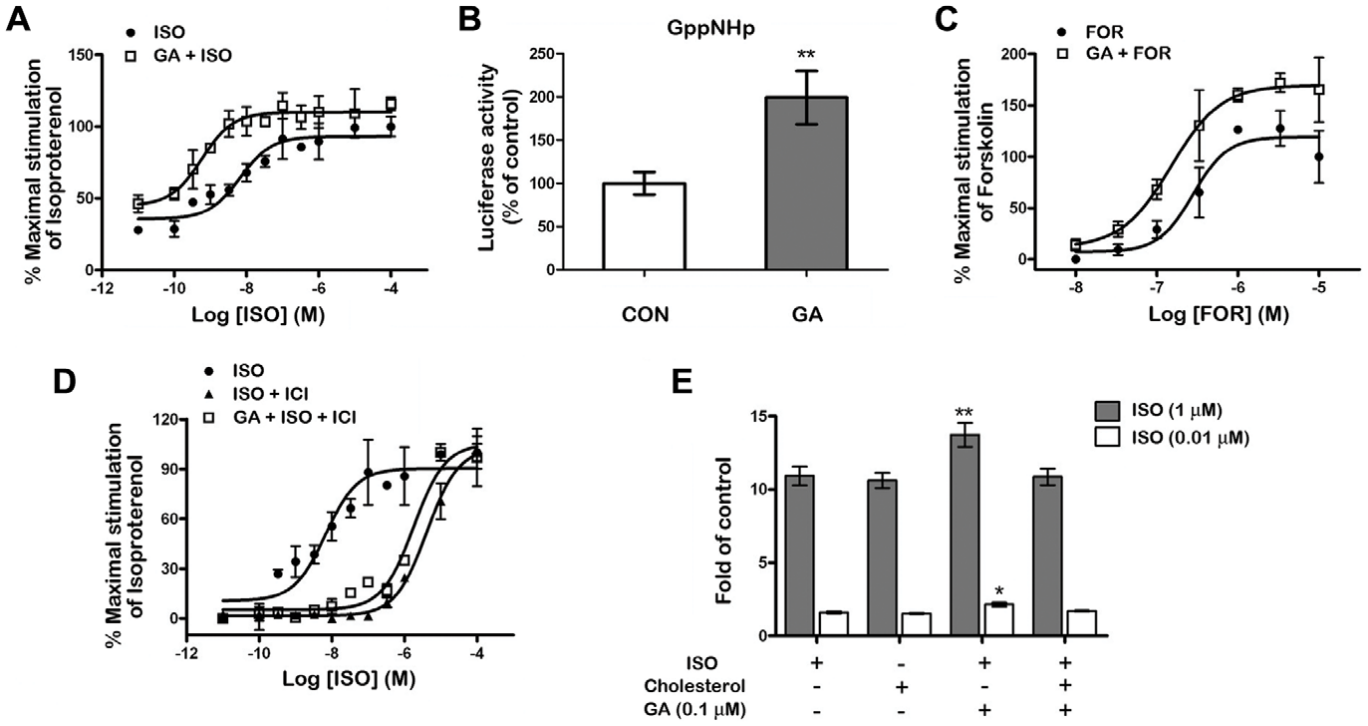
Effects of GA treatment on isoproterenol, forskolin, and GppNHp stimulated cAMP production. HA-β_2_AR-HEK293 cells pretreated with 0.1 µM GA or not were stimulated by isoproterenol (A), GppNHp (B) and forskolin (C). Detect of ICI 118 551 block effect were carry out with 0.1 µM GA pretreatment and 10 µM ICI 118 551 (D). Cholesterol (100 µM) were reloaded while 1 µM and 0.01 µM isoproterenol treatment (E). Further, the cAMP levels were determined by the cAMP- protein kinase A (PKA) reporter system in the graphs. Significant differences between the groups with or without cholesterol reloading were found. **means *p*<0.001; *means *p*<0.05, Student’s t test (*n* = 5).

To investigate the influence of GA on the internalization of β_2_ARs in live cells, the subcellular distribution of β_2_AR-EGFP was examined in HEK293 cells. As shown in Fig. 5, isoproterenol treatment induced β_2_ARs to move rapidly into punctate intracellular vesicles (Fig. 6A, 5 min to 30 min) with the appearance of early endosomes [37]. However, this phenomenon was repressed by a pretreatment of GA (Fig. 6B).

A quantitative ELISA assay was carried out for the quantitative determination of cell surface β_2_ARs. Treatment with isoproterenol for 2 h caused a maximal 21.11%±7.32% loss in cell surface β_2_ARs (Fig. 6C). In contrast, the GA pretreated group showed significantly lower loss. Co-immunoprecipitation of clathrin heavy chain with HA-β_2_AR also revealed that GA could decrease β_2_AR binding to clathrin (Fig. 4B, 15 min to 45 min), a necessary step in the internalization of β_2_ARs [38]. Taken together, GA inhibited the isoproterenol-induced β_2_AR internalization.

### GA-regulated β_2_AR Signaling Efficacy

Finally, to investigate whether or not GA influences the efficacy of the β_2_AR-Gα_s_-AC signaling cascade, cAMP levels were evaluated in HA-β_2_AR-HEK293 cells using the cAMP- protein kinase A (PKA) reporter system. Indeed, both basal and maximal isoproterenol-stimulated cAMP production increased after 0.1 µM GA pretreatment (Fig. 6A). To determine the specific site within the β_2_AR-Gα_s_-AC signaling cascade sensitized by 0.1 µM GA treatment, we further investigated functional responses to the non-hydrolysable GTP analogue 5-guanylimidodiphosphate (Gpp(NH)p) and to the AC agonist forskolin. Maximal Gpp(NH)p-stimulated cAMP production increased twofold after GA pretreatment (Fig. 6B), suggesting that a G protein may be involved in the GA-mediated response. Forskolin-stimulated cAMP production was also significantly increased, which is consistent with the notion that forskolin activates AC in a G protein-dependent manner (Fig. 6C) [39]. However, GA could not reverse the blocking effect of 10 µM ICI 118 551 on the β_2_ARs (Fig. 6D).

To test whether the signaling efficacy can be regulated by the GA induced cholesterol loss on membrane, we assessed the influence of cholesterol reloading on the β_2_AR signaling efficacy. Artificially increased membrane cholesterol concentration (100 µM) without prior GA treatment blocked the synergistic effect on β_2_AR signaling (Fig. 6E). This finding indicates that the changes in membrane cholesterol are related with β_2_AR-Gα_s_-AC signaling.

## Discussion

Earlier studies indicate that decreased membrane fluidity in the presence of glycyrrhizin could suppress the infectivity of HIV-1, influenza virus, and vesicular stomatitis virus [40], [41]. Recently, Schrofelbauer et al. demonstrated that glycyrrhizin attenuates pro-inflammatory responses by interfering with membrane-dependent receptor signaling [42]. These studies have led to the hypothesis that glycyrrhizin or GA may be incorporated into lipid bilayers to influence receptor signaling by altering the integrity and fluidity of the plasma membrane. In the present work, we treated cells with ATGA, azido-labeled GA, to assess cellular distribution. Based on both the biotin-streptavidin detection and 4-ethynyl-*N*-ethyl-1, 8-naphthalimide probe systems, most of the fluorescence was found to be concentrated at certain areas of the cell membrane and little was observed in the cytoplasm. Furthermore, a displacement effect of GA on cholesterol was detected by imaging of cholesterol location and sucrose gradient separation. Farther, the result of Spin-label ESR spectroscopy showed that GA treatment greatly influenced the hydrophobic regions of cell membranes but had little effect on the polar surface. Although GA has a rigid highly hydrophobic steroid ring structure, it lacks the bulky non-polar hydrocarbon tail of cholesterol. This feature could explain why GA increased the fluidity of the hydrophobic regions of the cell membranes but not the more polar surface domains. A previous study found that glycyrrhizin decreases membrane fluidity under 5-DSA labeling [40]. The influence of glycyrrhizin on the fluidity of the polar surface of cell membranes could be attributed to two more molecules of glucuronic acid at the polar head of GA. The decrease in *S*/*W*, which indicates a transition of many membrane proteins to a tighter tertiary conformation, may be caused by the GA-mediated formation of an active ternary complex with stronger sulfhydryl binding than in the inactive state.

Cholesterol plays an important function in membrane lipid raft. It is known to participate in various signal transductions. Changes in cholesterol content affect the localization of proteins in rafts [3], [43]. Based on lipid raft preparations, we established that β_2_ARs were excluded from lipid raft domains where were rich of Gα_s_. After GA treatment, we observed significant translocations of Gα_s_ from lipid raft fractions to the high-density fractions. However, minimal changes were observed in the raft levels of flotillins and caveolin-1. These results indicate that the influences of GA on the localization of different signaling molecules are unique, likely reflecting different forms of chemical association with the membrane.

The enhanced coupling of β_2_ARs and Gα_s_ after treatment with GA suggests that GA influences the efficiency of receptor activation. Currently, the most widely accepted model for GPCR activation is the extended ternary complex model (the two-state model) [44], [45]. This model proposes that the receptor exists in an equilibrium between an inactive conformation (R) and an active conformation (R*) that is coupled to the G protein. In the absence of an agonist, the inactive R state prevails. However, the energy barrier between the R and R* states is sufficiently low to allow a certain fraction of the receptors to spontaneously assume the R* state. Agonists are predicted to bind with higher affinity to the R* conformation and consequently shift the equilibrium as well as increase the proportion of receptors in R*. In this paper, GA treatment increased the opportunity of molecular collisions between β_2_ARs and Gαs to increase through interaction with cholesterol, as well as by releasing a sequestered pool of Gα_s_ from lipid rafts that become available for β_2_ARs engagement. The increased β_2_ARs/Gα_s_ coupling promotes an increase in the active conformation (R*) of β_2_AR, thereby leading to increased cAMP responses to β2AR agonists. In this study, GA was also found increases the maximal Gpp(NH)p and forskolin-stimulated cAMP production, as Gα_s_ was found embedded in lipid raft domains and be excluded under GA treatment, this observation indicated that lipid rafts appear to play a negative regulatory role in β_2_AR signal transductions. Indeed, cholesterol depletion has been found to increase β_2_AR-stimulated cAMP production [20], [21], whereas cholesterol supplementation has an inverse effect [22]. This finding was also confirmed by our present data. Cholesterol reloading significantly attenuated the effect of GA on agonist-stimulated cAMP production, further suggesting that GA interacts with cholesterol in lipid rafts.

In contrast, several studies established that β_2_ARs undergo rapid phosphorylation by both second messenger dependent protein kinases and GPCR kinases in response to agonist stimulation [38]. This event targets receptors for binding to arrestin proteins that uncouple receptors from their cognate heterotrimeric G proteins, and favor receptor endocytosis via clathrin-coated vesicles into endosomal compartments [46], [47]. Our results demonstrate that GA could significantly inhibit isoproterenol-induced β_2_AR internalization, consistent with a previous study demonstrating that saponins could inhibit receptor internalization due to their cholesterol-complexing characteristics [48]–[51]. We speculate that the constant coupling between β_2_AR and Gα_s_ allows the receptor to maintain a sterical conformation not suitable for the binding of protein kinases and ensuing arrestin proteins. This speculation could also explain the GA-mediated reduction in the association between clathrin and β_2_AR. Di Certo et al. demonstrated that the fusion of β_2_AR to Gα_s_ slows agonist-induced internalization and strongly affects the recycling of receptors to the plasma membrane [52]. Bertin et al. reported that a β_2_AR/Gα_s_ fusion protein is more resistant to desensitization and enhances the anti-proliferative effect of isoproterenol in transfected S49 cells [53].

Taken together, we speculate that unlike β_2_ARs themselves, a significant fraction of their signaling partners is embedded in lipid raft microdomains of the membrane. GA can decrease the cholesterol content in lipid rafts and change the fluidity of the cell membrane, resulting in the release of raft-embedded Gα_s_ as well as increased interaction with β_2_ARs, thereby increasing β_2_AR/Gα_s_ coupling and decreasing receptor internalization. Ultimately, β_2_AR-mediated signal transduction is enhanced.

## Supporting Information

Materials S1 **Synthesis of azide-terminal glycyrrhetic acid.**
(DOC)

Materials S2 **Synthesis of 4-ethynyl-N-ethyl-1, 8– naphthalimide.**
(DOC)

Materials S3 **Synthesis of biotin-LC-alkyne.**
(DOC)
