# Supplementary material for: Glycyrrhetic Acid Synergistically Enhances β2-Adrenergic Receptor-Gs Signaling by Changing the Location of Gαs in Lipid Rafts
Source: PLoS One. 2012 Sep 27;7(9):e44921. doi: 10.1371/journal.pone.0044921 (PMC3459958; doi:10.1371/journal.pone.0044921)
Supplement: Materials S1 — Synthesis of azide-terminal glycyrrhetic acid. (DOC) [file pone.0044921.s001.doc]

**Materials S1**: Synthesis of azide-terminal glycyrrhetic acid

Glycyrrhetic acid (1 g, 2.12 mmol) was dissolved in 15 ml of pyridine at 0°C, and then 10 ml of acetic anhydride was added drop wise. The mixture was stirred at room temperature for 5 h and then poured into ice water, resulting in a white solid precipitate. The crude product was dissolved in 20 ml chloroform, and then 7 ml thionyl chloride was added drop wise. The mixture was stirred at 0°C for 0.5 h, and then refluxed for 3 h under 70 °C. The solution was poured into ice water. The solidified mass was dissolved in 15 ml dichloromethane and 1 ml pyridine and 3-chloropropylamine hydrochloride (89.5 mg, 0.77 mmol) were added sequentially. The mixture was stirred at 0°C for 2 h, and then washed with water, saturate sodium carbonate solution, and brine sequentially. The organic phase was dried by anhydrous magnesium sulphate and then evaporated under reduced pressure to yield a white solid (70% yield by weight). The compound (70 mg) was dissolved in 10 ml DMF, and NaN_3_ (15.3 mg, 0.236 mmol) was slowly added to the solution under stirring at 60°C for 10 h. The mixture was precipitated in ice water and filtered to yield a white powder. This compound was dissolved in methanol, and 1 M CH_3_ONa in methanol was dripped into the solution and the new mixture stirred at room temperature for 4 h. After this reaction, the mixture was washed with water, citric acid and, brine. The organic phase was dried by anhydrous magnesium sulphate and then evaporated under reduced pressure. The residue was purified by a silica gel column (30% ethyl acetate-petroleum ether) to afford a white solid with 80% yield. ^1^H-NMR(CDCl_3_, 400 MHz), 2.82 (37, 1H, dd, J=8.990, J=5.590), 1.35 (38, 1H, dd, J=5.350, J=1.710), 0.91 (3, 3H), 1.02 (4, 3H), 1.17 (5, 3H), 0.893 (6, 3H), 1.28 (7, 3H), 0.94 (8, 3H), 0.99 (9, 3H), 6.16 (14, 1H), 4.68 (15, 2H, t, J=7.386), 3.54 (16, 2H, t, J=7.386), 1.96 (17, 1H, dddd, J=10.634, J=3.070, J=3.000, J=2.580), 2.05 (17, 1H, dddd, J=10.634, J=10.240, J=2.460, J=1.910), 1.71 (18, 1H, ddd, J=12.920, J=4.550, J=2.180), 1.56 (18, 1H, ddd, J=12.920, J=9.500, J=3.760), 1.81 (19, 1H, ddd, J=14.179, J=3.760, J=2.180), 1.55 (19, 1H, ddd, J=14.179, J=9.500, J=4.550), 1.68 (20, 1H, ddd, J=13.128, J=4.060, J=2.080), 1.49 (20, 1H, ddd, J=13.128, J=9.540, J=4.360), 1.83 (21, 1H, ddd, J=13.223, J=2.580, J=2.460), 1.58 (21, 1H, ddd, J=13.223, J=10.240, J=3.070), 1.84 (22, 1H, dddd, J=13.398, J=4.350, J=2.220, J=1.710), 1.51 (22, 1H, dddd, J=13.398, J=8.950, J=5.350, J=4.600), 1.66 (23, 1H, ddd, J=13.112, J=4.360, J=2.080), 1.50 (23, 1H, ddd, J=13.112, J=9.540, J=4.060), 1.49 (24, 1H, ddd, J=13.044, J=4.600, J=2.220), 1.53 (24, 1H, ddd, J=13.044, J=8.950, J=4.350), 2.02 (25, 1H, dd, J=13.374, J=5.590), 1.93 (25, 1H, dd, J=13.374, J=8.990), 3.52 (32, 1H, dd, J=3.000, J=1.910), 2.90 (33, 1H).
