# Supplementary material for: Glycyrrhetic Acid Synergistically Enhances β2-Adrenergic Receptor-Gs Signaling by Changing the Location of Gαs in Lipid Rafts
Source: PLoS One. 2012 Sep 27;7(9):e44921. doi: 10.1371/journal.pone.0044921 (PMC3459958; doi:10.1371/journal.pone.0044921)
Supplement: Materials S2 — Synthesis of 4-ethynyl-N-ethyl-1, 8– naphthalimide. (DOC) [file pone.0044921.s002.doc]

**Materials S2**: Synthesis of 4-ethynyl-N-ethyl-1, 8– naphthalimide

4-bromo-*N*-ethyl-4-ethynyl-N-ethyl-1,8-naphthalimide (234 mg, 0.77 mmol) was dissolved in 10 ml of THF, and then tetrakis (triphenylphosphine) palladium (90 mg, 0.078 mmol), CuI (30 mg, 0.16 mmol), trimethylsilylacetylene (0.54 ml, 3.82 mmol), and *N*, *N*-diisopropylethylamine (0.5 ml, 2.87 mol) were added under argon gas. The mixture was stirred at room temperature overnight. The reaction mixture was diluted with AcOEt, washed with saturated NH_4_Cl solution, dried over Na_2_SO_4_, and evaporated. The residue was purified partially by flash column chromatography on silica gel (AcOEt/hexane 1:10) to yield the corresponding trimethylsilyl compound (180 mg). To a solution of this compound (180 mg) in 25 ml of MeOH was added 1 M tetrabutylammonium fluoride solution in THF (2 ml, 2 mmol), and the mixture was stirred at 60 °C for 15 min. The reaction mixture was diluted with water, and the precipitates were collected by filtration. The solids were purified by flash column chromatography on silica gel (AcOEt/hexane 1:5) to yield a colourless solid (65 mg, 34%). ^1^H-NMR (500 MHz, CDCl_3_), 1.34 (t, 3H, *J* = 7.0 Hz), 3.73 (s, 1H), 4.25 (q, 2H, *J* = 7.0 Hz), 7.83 (m, 1H), 7.94 (d, 1H, *J* = 7.5 Hz), 8.54 (d, 1H, *J* = 7.3 Hz), 8.64 (d, 1H, *J* = 7.5 Hz), 8.67 (d, 1H, *J* = 8.5 Hz); ESI-TOF-HRMS *m/e* calculated for (M+H)^+^ C_16_H_12_NO_2_ 250.0863; found 250.0866.
