# Supplementary material for: Glycyrrhetic Acid Synergistically Enhances β2-Adrenergic Receptor-Gs Signaling by Changing the Location of Gαs in Lipid Rafts
Source: PLoS One. 2012 Sep 27;7(9):e44921. doi: 10.1371/journal.pone.0044921 (PMC3459958; doi:10.1371/journal.pone.0044921)
Supplement: Materials S3 — Synthesis of biotin-LC-alkyne. (DOC) [file pone.0044921.s003.doc]

**Materials S3**: Synthesis of biotin-LC-alkyne


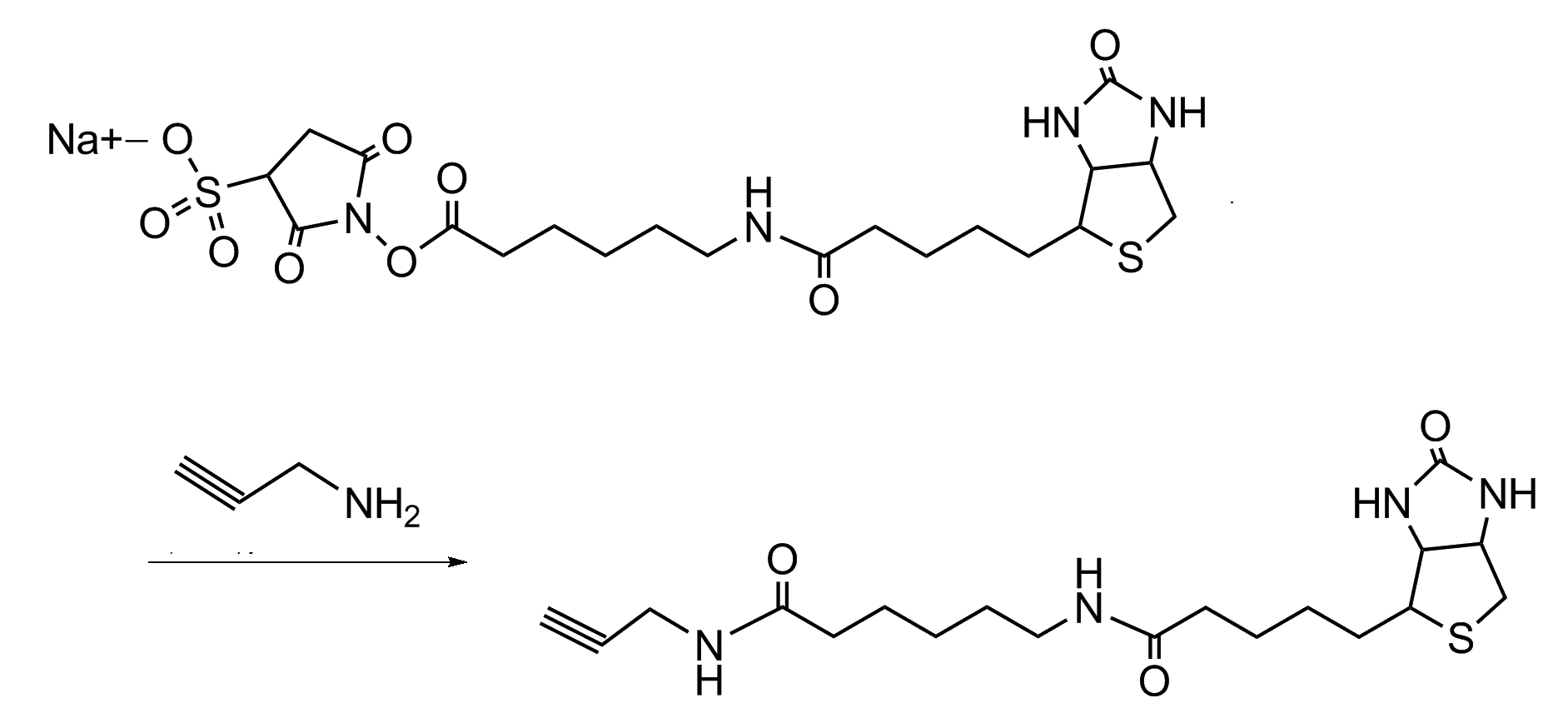


Sulfo-NHS-LC-Biotin (20 mg, 0.036 mmol, Thermo Scientific Pierce) was dissolved in 0.2 ml of borate buffer (0.2 M pH 8) and then 2 μl propargylamine was added. The mixture was stirred at room temperature for 4 h resulting in a white solid precipitate. The microscale crude product was collected by centrifugation and dissolved in 50 μl methanol and the purified by chromatography silica gel plate (chloroform/methanol 5:1) to afford a white solid with 75% yield.
